# Harnessing longitudinal information to identify genetic variation in tolerance of pigs to Porcine Reproductive and Respiratory Syndrome virus infection

**DOI:** 10.1186/s12711-018-0420-z

**Published:** 2018-10-24

**Authors:** Graham Lough, Andrew Hess, Melanie Hess, Hamed Rashidi, Oswald Matika, Joan K. Lunney, Raymond R. R. Rowland, Ilias Kyriazakis, Han A. Mulder, Jack C. M. Dekkers, Andrea Doeschl-Wilson

**Affiliations:** 1 The Roslin Institute and R(D)SVS, University of Edinburgh, Edinburgh, Midlothian UK; 2 Department of Animal Science, Iowa State University, Ames, IA 50011 USA; 3 Animal Breeding and Genomics, Wageningen University and Research, PO Box 338, 6700 AH Wageningen, The Netherlands; 4 School of Agriculture Food and Rural Development, Newcastle University, Newcastle upon Tyne, NE1 7RU UK; 5 Animal Parasitic Diseases Laboratory, BARC, ARS, USDA, Beltsville, MD 20705 USA; 6 College of Veterinary Medicine, Kansas State University, Manhattan, KS 66506 USA

## Abstract

**Background:**

High resistance (the ability of the host to reduce pathogen load) and tolerance (the ability to maintain high performance at a given pathogen load) are two desirable host traits for producing animals that are resilient to infections. For Porcine Reproductive and Respiratory Syndrome (PRRS), one of the most devastating swine diseases worldwide, studies have identified substantial genetic variation in resistance of pigs, but evidence for genetic variation in tolerance has so far been inconclusive. Resistance and tolerance are usually considered as static traits. In this study, we used longitudinal viremia measurements of PRRS virus infected pigs to define discrete stages of infection based on viremia profile characteristics. These were used to investigate host genetic effects on viral load (VL) and growth at different stages of infection, to quantify genetic variation in tolerance at these stages and throughout the entire 42-day observation period, and to assess whether the single nucleotide polymorphism (SNP) WUR10000125 (WUR) with known large effects on resistance confers significant differences in tolerance.

**Results:**

Genetic correlations between resistance and growth changed considerably over time. Individuals that expressed high genetic resistance early in infection tended to grow slower during that time-period, but were more likely to experience lower VL and recovery in growth by the later stage. The WUR genotype was most strongly associated with VL at early- to mid-stages of infection, and with growth at mid- to late-stages of infection. Both, single-stage and repeated measurements random regression models identified significant genetic variation in tolerance. The WUR SNP was significantly associated only with the overall tolerance slope fitted through all stages of infection, with the genetically more resistant AB pigs for the WUR SNP being also more tolerant to PRRS.

**Conclusions:**

The results suggest that genetic selection for improved tolerance of pigs to PRRS is possible in principle, but may be feasible only with genomic selection, requiring intense recording schemes that involve repeated measurements to reliably estimate genetic effects. In the absence of such records, consideration of the WUR genotype in current selection schemes appears to be a promising strategy to improve simultaneously resistance and tolerance of growing pigs to PRRS.

**Electronic supplementary material:**

The online version of this article (10.1186/s12711-018-0420-z) contains supplementary material, which is available to authorized users.

## Background

Infectious diseases pose a considerable problem for livestock production. Where conventional control strategies, such as culling or vaccination, have limited success in controlling or eliminating an infectious agent, genetic selection for improved host response to infection challenge may be an attractive solution [1].

In livestock production, disease resilience, defined as the ability of a host to maintain high production levels when challenged with infectious pathogens [2], has become a desirable breeding goal trait [3, 4]. Disease resilience comprises two alternative defence mechanisms under potential host genetic control: resistance, which is the ability of the host to inhibit or reduce pathogen replication [5–8]; and tolerance, which is the ability of the host to limit the impact of the infection on host performance, without necessarily affecting pathogen burden [9–12]. Hence, both tolerance and resilience measure how performance is affected by infectious challenge, but resilience does not take differences in within-host–pathogen burden (i.e. resistance) into account, whereas tolerance measures performance at given within-host–pathogen burden. Both resilience components, i.e. resistance and tolerance may harbour substantial genetic variation and covariation, and are thought to have different impacts on disease epidemiology and pathogen evolution [13, 14]. Furthermore, a recent theoretical study has shown that inclusion of both resistance and tolerance in the selection objective can lead to higher genetic gain in performance under pathogen challenge than selection on resilience [15]. Thus, identifying and quantifying genetic variation (and co-variation) in both these traits, host resistance and tolerance, is crucial for devising effective breeding programmes that maintain high health and production levels in the face of infectious challenges. Numerous disease challenge experiments have provided ample evidence for considerable genetic variance in host resistance [16]. In contrast, estimates for genetic variance in tolerance of farm animals to infections are still sparse, in part because tolerance is defined as the slope of reaction-norms of performance against within-host–pathogen burden [17], for which reliable variance component estimates are difficult to obtain [18].

Genetic improvement of host resistance and tolerance has been considered as a viable disease control strategy for Porcine Reproductive and Respiratory Syndrome (PRRS) [13]. PRRS is an endemic viral disease that causes respiratory problems and considerable reduction in piglet growth rate and sow reproductive performance [19–21], and has led to severe economic losses to the swine industry in countries worldwide [22, 23]. Large-scale PRRS virus (PRRSV) challenge studies carried out by the PRRS Host Genetics Consortium (PHGC) have demonstrated considerable genetic variation in resistance of growing piglets to PRRSV infection based on viral load (VL) (within-host VL, defined as cumulative log viremia until 21 days post-infection), as well as in growth under infection, regardless of VL (i.e. resilience) [24–26]. Furthermore, using the same datasets, a quantitative trait locus (QTL) was identified on chromosome 4, where the single nucleotide polymorphism (SNP) WUR10000125 (WUR) explained 13.2 and 9.1% of the genetic variance for VL and growth, respectively [24–26]. Pigs that were heterozygous *AB* for the WUR locus had on average 4.5% lower VL and grew on average 2 kg more over the 42-day infection period than individuals with the unfavourable *AA* genotype. Our previous study of the same PHGC data found inconclusive evidence for genetic variation in tolerance of pigs to PRRS, which was attributed to limited statistical power to disentangle genetic effects related to the overall growth response under infection, regardless of VL and those related to tolerance [10]. This previous study, which was based on VL and growth as single cumulative measures for each individual over time periods of 21 or 42 days, concluded that more measurements, in particularly a wider spread of individual VL measurements for fitting regression curves (e.g. including zero VL), would potentially resolve this confounding and may permit accurate estimation of genetic variance in tolerance [10].

The hypothesis of the current research was that significant genetic variance in tolerance of pigs to PRRS exists and can be detected by partitioning the infection period into distinct stages of infection. This harnesses available longitudinal information of pigs’ responses to infection and builds on several independent studies, which indicated that tolerance changes over the time-course of infection [11, 12, 27, 28]. Hence, variance estimates for tolerance may also change over time. The specific aims of this research were to estimate genetic variation in tolerance at different stages following experimental infection with PRRSV, defined by viremia curve characteristics, and throughout the whole 42-day observation period, by using the PHGC data from the different infection stages in repeated measures models. Furthermore, this study investigated whether the previously identified WUR SNP, which is associated with resistance and growth under infection, was also associated with tolerance, and whether the strength of the association of the WUR SNP with resistance and growth changed during the course of the infection.

## Methods

### Infection experiment and data

The PRRS Host Genetics Consortium (PHGC) dataset used in this study was the same as that of a previous study that provided inconclusive evidence for genetic variation in tolerance [10]. Briefly, data from 1569 commercial crossbred growing pigs supplied by various breeding companies were collected from nine PRRSV challenge trials following an identical infection protocol [29]. The animal composition in each of these trials is in Additional file 1: Table S1. At weaning age (mean age was 26 days, and ages ranged from 17 to 32 days), around 200 piglets in each trial were transferred from farms (free of PRRSV, *Mycoplasma hyopneumoniae*, and swine influenza virus) to a research facility at Kansas State University. Pigs were randomly assigned to pens of between 10 and 15 individuals. After a 7-day acclimation period, pigs were infected both intranasally and intramuscularly with 10^5^ (TCID50) of NVSL-97-7985, a highly virulent PRRSV type-2 isolate [30]. Data were collected for body weight (BW) weekly and from blood samples at 0, 4, 7, 11, 14, 21, 28, 35 and 42 days-post-infection (dpi). At 42 dpi, pigs were euthanized and ear notches were collected for genotyping. Due to facility availability, trials 7 and 8 were terminated at 35 dpi. As described in Boddicker et al. [24], serum virus load, measured by a semi-quantitative TaqMan PCR assay for PRRSV RNA, provided repeated measures for log_10_-transformed qPCR viremia.

Across all trials, 198 pigs died before 42 dpi. The primary cause of mortality was attributed to PRRS, except for trial 6, which had 46% higher mortality than the other trials due to secondary bacterial infections [31]. Nevertheless, data from these pigs prior to death were also included in the analyses [6, 11, 24, 31].

Pedigree and genomic information from Illumina’s Porcine SNP60 Beadchip v.1 [32] was known for all pigs. The pedigree-based numerator relationship matrix (${\mathbf{A}}$) was constructed in ASReml 3.0 [33]. Each pig was assigned to one of three WUR genotypes, using the Illumina *A*/*B* genotype reference system (*AA* = 689, *AB* = 286 and *BB* = 36, where allele *B* was known to confer higher growth and lower VL).

As in the previous tolerance study [10], only offspring from sires with more than 10 progeny with phenotypes were considered to reduce the risk of bias in tolerance estimates [17]. As such, the number of animals included was 1001 from 49 sires. In contrast to our previous study [10], 214 pigs that had experienced a rebound in viremia (i.e. a second period of increased serum virus load), as identified by curve fitting [34], were omitted from the analyses. Hence, all pigs considered in this study experienced a gradual viremia decline after peak viremia had been reached. This allowed the 42-day infection period to be partitioned into three distinct stages based on common viremia curve characteristics, as described in the next section.

### Defining stages of infection

**Fig. 1:**
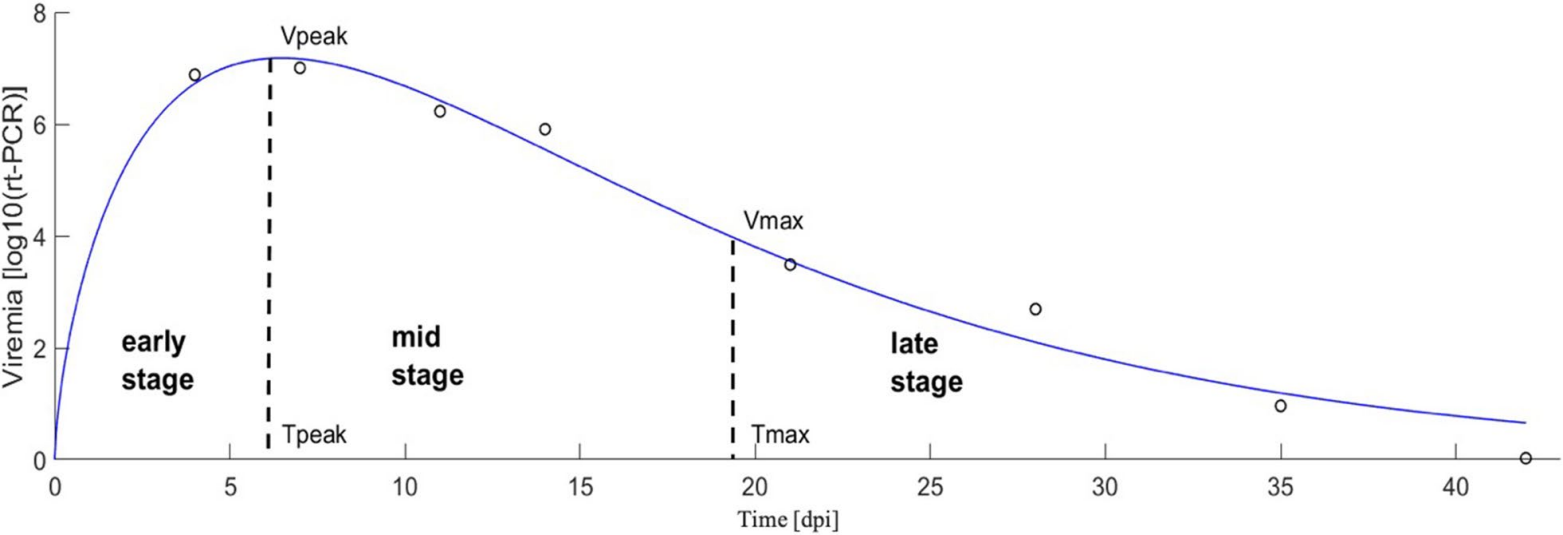
Illustration of an individual viremia profile used to define stages of infection. Black dots represent log-transformed viremia measures, and the blue line represents the fitted Woods function over the time-course of infection (up to 42 dpi) for one individual. Early-stage of infection is defined from initial infection (0 dpi) to time when peak viremia (${\text{V}}_{\rm{peak}}$) is reached (${\text{T}}_{\rm{peak}}$ in this example, approximately 6 dpi); mid-stage of infection is defined from ${\text{T}}_{\rm{peak}}$ to ${\text{T}}_{ \rm{max} }$ (${\text{T}}_{ \rm{max} }$ in this example, approximately 19 dpi); and late-stage of infection, defined from ${\text{T}}_{ \rm{max} }$ to end of experiment (42 dpi, or 35 dpi for trials 7 and 8)

In contrast to most previous genetic studies of these PHGC data, which defined resistance and tolerance over 0 to 21 or 0 to 42 dpi periods [6, 10, 24, 31], in this study the experimental observation period was partitioned into three stages, i.e. early-, mid- and late-stage of infection. Stages of infection were defined individually based on viremia profile characteristics obtained by fitting the mathematical Woods function described below to the repeated viremia measures of each individual pig, as illustrated in Fig. 1 [26, 34]. More specifically, the Wood’s function, which had previously been shown to be a good fit for the viremia data of an individual pig $i$ that did not experience viremia rebound [34], is given by $V_{i} \left( t \right) = a_{i} t^{{b_{i} }} e^{{ - c_{i} t}}$, where $V_{i} \left( t \right)$ is the log_10_ scaled serum viremia of pig $i$ at $t$ dpi; $a_{i}$ is a scaler quantity which defines the overall height of the curve; $b_{i}$ governs the rate of increase to peak viremia, and parameter $c_{i}$ is an indicator of the rate of decline after peak viremia. Using the analytical expression for $\left( t \right)$, two critical time points can be derived by differentiation: ${\text{T}}_{{{\rm{peak}}_{i} }} = \frac{{\hat{b}_{i} }}{{{\hat{\text{c}}}_{\rm{i}} }}$, the time when viremia reaches its maximum value and $T_{{{\rm max}_{i} }} = \frac{{\hat{b}_{i} + \sqrt {\hat{b}_{i} } }}{{{\hat{\text{c}}}_{\rm{i}} }}$, the time post-peak viremia when the rate of viremia decline reaches its maximum (i.e. the inflection point of the Woods function). These critical time points (${\text{T}}_{\rm{peak}}$ and ${\text{T}}_{ \rm{max} }$, respectively) were used to define the lower and upper boundaries of each stage of infection in this study. Thus, stages of infection were defined for each individual from time of initial infection (0 days post-infection (dpi)) to ${\text{T}}_{{{\rm{peak}}_{i} }}$ (early); from ${\text{T}}_{{{\rm{peak}}_{i} }}$ to $T_{{max_{i} }}$ (mid), and from $T_{{max_{i} }}$ to the end of the observed infection period, which was at 35 dpi for trials 7 and 8, and 42 dpi for all other trials (late). This way, the early-stage corresponds to the phase of rapid viremia increase towards individual peak viremia, the mid-stage corresponds to the initial phase of rapid post-peak viremia decline, and the late-stage corresponds to the phase where viremia continues to decline at a decreasing rate.

Since the Woods function was fitted to each individual separately [34], estimates for the parameters ${\text{T}}_{\rm{peak}}$ and ${\text{T}}_{ \rm{max} }$ were obtained for each individual. Table 1 shows the variation in the corresponding time-periods for each stage of infection. Average duration of each stage of infection was 6.90, 9.19 and 14.26 days for early-, mid- and late-stages of infection, respectively, corresponding to average ${\text{T}}_{\rm{peak}}$ and ${\text{T}}_{ \rm{max} }$ of 6.90 and 16.16 days, respectively.

**Table 1:** Descriptive statistics of duration (days) for each stage of infection

Stage of infection	Mean	Standard deviation	Minimum	Maximum
Early	6.90	1.32	3.01	10.60
Mid	9.19	2.11	4.04	14.87
Late	14.26	0.60	13.53	14.92
${\text{T}}_{\rm{peak}}$	6.90	1.32	3.01	10.60
${\text{T}}_{ \rm{max} }$	16.16	2.38	10.01	26.97

Early-stage is defined as initial infection (0 dpi) to ${\text{T}}_{\rm{peak}}$; mid-stage is defined as ${\text{T}}_{\rm{peak}}$ to ${\text{T}}_{ \rm{max} }$, and late-stage of infection is defined from ${\text{T}}_{ \rm{max} }$ to end of the observation period (35 dpi for trials 7 an 8 and 42 dpi for all other trials). Descriptive statistics of ${\text{T}}_{\rm{peak}}$ and ${\text{T}}_{ \rm{max} }$ are also shown. All measures are in days

In line with previous studies, resistance during a particular stage of infection was then quantified as the inverse of the cumulative log-transformed VL over the corresponding time-period [9, 10, 18, 26]. This was calculated as the area under the curve of the log-transformed viremia estimates throughout the observation period, obtained by numerical integration of the Woods function over the corresponding time-periods, yielding estimates for VL at early-, mid- and late-stages of infection.

To calculate the corresponding ADG for each stage of infection, a linear spline curve was fitted through weekly body weights (BW) for each individual using the *smooth.spline* function in R. BW was interpolated at time of peak viremia (${\text{T}}_{\rm{peak}}$) and time of maximal viremia decay (${\text{T}}_{ \rm{max} }$) using the *predict* function in R. From this, ADG at early-, mid- and late-stages of infection were calculated by dividing the difference in smoothed body weight at the start and end of the stage in consideration by the corresponding duration.

### Statistical analyses

All statistical analyses were carried out using ASReml 3.0 [33], with genetic relatedness described by the pedigree relationship matrix for consistency with our previous tolerance analysis of the same dataset [10, 33]. Replacing the pedigree relationship matrix with a genomic relationship matrix (which can capture differences between siblings due to Mendelian sampling) had only negligible impact on the variance estimates and on the goodness of fit statistics (results not shown).

### Estimating the genetic correlation between ADG and VL within and between stages of infection

The first step in the analysis of the variation in resistance and tolerance to infection was to estimate heritabilities and correlations between VL and growth at each stage of infection using a conventional bi-variate animal model for VL and ADG for all individual stages of infection:

1 $$\left[ {\begin{array}{*{20}c} {{\mathbf{y}}_{i} } \\ {{\mathbf{y}}_{j} } \\ \end{array} } \right] = \left[ {\begin{array}{*{20}c} {{\mathbf{X}}_{1} } & 0 \\ 0 & {{\mathbf{X}}_{2} } \\ \end{array} } \right]\left[ {\begin{array}{*{20}c} {{\mathbf{b}}_{1} } \\ {{\mathbf{b}}_{2} } \\ \end{array} } \right] + \left[ {\begin{array}{*{20}c} {{\mathbf{Z}}_{1} } & 0 \\ 0 & {{\mathbf{Z}}_{2} } \\ \end{array} } \right]\left[ {\begin{array}{*{20}c} {{\mathbf{a}}_{1} } \\ {{\mathbf{a}}_{2} } \\ \end{array} } \right] + \left[ {\begin{array}{*{20}c} {{\mathbf{U}}_{1} } & 0 \\ 0 & {{\mathbf{U}}_{2} } \\ \end{array} } \right]\left[ {\begin{array}{*{20}c} {{\mathbf{p}}_{1} } \\ {{\mathbf{p}}_{2} } \\ \end{array} } \right] + \left[ {\begin{array}{*{20}c} {{\mathbf{M}}_{1} } & 0 \\ 0 & {{\mathbf{M}}_{2} } \\ \end{array} } \right]\left[ {\begin{array}{*{20}c} {{\mathbf{l}}_{1} } \\ {{\mathbf{l}}_{2} } \\ \end{array} } \right] + \left[ {\begin{array}{*{20}c} {{\mathbf{e}}_{1} } \\ {{\mathbf{e}}_{2} } \\ \end{array} } \right],$$

where ${\mathbf{y}}_{{\mathbf{i}}}$ and ${\mathbf{y}}_{{\mathbf{j}}}$ are vectors of phenotypes for ADG (${\mathbf{y}}_{{\mathbf{i}}}$), and VL (${\mathbf{y}}_{{\mathbf{j}}}$), respectively, at the stages ${\text{i}}$ and ${\text{j}}$ of infection (e.g. ADG at stage ${\text{i}}$, { ${\text{i}}$ = early, mid, late} and VL at stage ${\text{j}}$, { ${\text{j}}$ = early, mid, late}); ${\mathbf{b}}_{1}$ and ${\mathbf{b}}_{2}$ are the vectors of the trait means and fixed effects for the interaction of experimental trial and parity of the dam when offspring were born (trial-by-parity), sex of the offspring, BW and age at the start of experimental infection included as additional fixed covariates to account for body weight at the initial infection and variation in age at the start of infection, respectively. Note that no breed effect was included in the model since trial and breed effects were fully confounded in this experiment; ${\mathbf{a}}_{1}$, and ${\mathbf{a}}_{2}$ are the vectors of additive genetic effects for each trait, with $${\text{Var}}\left[ {\begin{array}{*{20}c} {{\mathbf{a}}_{1} } \\ {{\mathbf{a}}_{2} } \\ \end{array} } \right] = {\mathbf{G}} \otimes {\mathbf{A}}$$, where ${\mathbf{G}}$ is the genetic variance–covariance matrix and ${\mathbf{A}}$ the pedigree relationship matrix; ${\mathbf{p}}_{1}$ and ${\mathbf{p}}_{2}$ are the vectors of pen effects nested within a trial for each trait, with $${\text{Var}}\left[ {\begin{array}{*{20}c} {{\mathbf{p}}_{1} } \\ {{\mathbf{p}}_{2} } \\ \end{array} } \right] = {\mathbf{I}} \otimes {\mathbf{K}}$$, where **I** is the identity matrix and ${\mathbf{K}}$ is the corresponding variance–covariance matrix of pen effects for the different traits; ${\mathbf{l}}_{1}$ and ${\mathbf{l}}_{2}$ are the vectors of litter effects for each trait, with $${\text{Var}}\left[ {\begin{array}{*{20}c} {{\mathbf{l}}_{1} } \\ {{\mathbf{l}}_{2} } \\ \end{array} } \right] = {\mathbf{I}} \otimes {\mathbf{L}}$$, with the corresponding variance–covariance matrix ${\mathbf{L}}$; ${\mathbf{e}}_{1}$, and ${\mathbf{e}}_{2}$ are the vectors of error terms for each trait, with $${\text{Var}}\left[ {\begin{array}{*{20}c} {{\mathbf{e}}_{1} } \\ {{\mathbf{e}}_{2} } \\ \end{array} } \right] = {\mathbf{I}} \otimes {\mathbf{R}}$$, where ${\mathbf{R}}$ is the variance–covariance matrix for the residual effects for each trait; and ${\mathbf{X}}_{1}$ and ${\mathbf{X}}_{2}$, ${\mathbf{Z}}_{1}$ and ${\mathbf{Z}}_{2}$, ${\mathbf{U}}_{1}$ and ${\mathbf{U}}_{2}$, and ${\mathbf{M}}_{1}$ and ${\mathbf{M}}_{2}$ are the incidence matrices for the fixed, animal, pen and litter effects, respectively. In addition to the bivariate animal model, corresponding univariate models were also used to check the robustness of variance components. Since heritability estimates differed between models, heritability estimates from the univariate models are presented.

### Estimation of the genetic variance in tolerance at each stage of infection

To estimate genetic variance in tolerance, a random regression reaction-norm model was applied to the data. Information on performance in absence of infection was unavailable. To account for this, the origin of the reaction-norms (intercept) was centred at the mean VL for each stage of infection across all animals, referred to as ‘level.’ As described in a previous study [10], the linear random regression sire model (RRM) for ADG on centred values of VL, which will be referred to as the level-slope model, was used to identify genetic variance in tolerance at each stage of infection, where ADG at early-, mid- and late-stages of infection were regressed either on VL at the corresponding or previous stages of infection (for example ADG at late-stage of infection regressed on VL at mid-stage of infection):

2 $${\mathbf{y}} = {\mathbf{Xb}} + {\mathbf{X}}_{{{\mathbf{VL}}}} {\mathbf{b}}_{{\mathbf{s}}} + {\mathbf{Za}}_{{\mathbf{i}}} + {\mathbf{Z}}_{{{\mathbf{VL}}}} {\mathbf{a}}_{{\mathbf{s}}} + {\mathbf{Up}} + {\mathbf{Ml}} + {\mathbf{e}},$$

where ${\mathbf{y}}$ is the vector of ADG at early-, mid- or late-stages of infection; ${\mathbf{b}}$ is the vector of fixed effects described in model (1), where level (overall mean at centred VL) is fixed; and ${\mathbf{b}}_{\varvec{s}}$ is the population average tolerance slope (fixed VL); ${\mathbf{a}}_{\varvec{i}}$ and ${\mathbf{a}}_{\varvec{s}}$ are the sire effects on level (intercept at centred VL) and on tolerance slope, respectively, assumed to follow a multi-variate normal distribution with a mean of zero and $${\text{Var}}\left[ {\begin{array}{l} {{\mathbf{a}}_{{\mathbf{i}}} } \\ {{\mathbf{a}}_{{\mathbf{s}}} } \\ \end{array} } \right] = \frac{1}{4}{\mathbf{G}} \otimes {\mathbf{A}}$$, with $${\mathbf{G}} = \left[ {\begin{array}{ll} {\upsigma_{{{\rm{a}}_{\rm{i}} }}^{2} } & {\upsigma_{{{\rm{a}}_{\rm{i}} {\rm{a}}_{\rm{s}} }} } \\ {\upsigma_{{{\rm{a}}_{\rm{i}} {\rm{a}}_{\rm{s}} }} } & {\upsigma_{{{\rm{a}}_{\rm{s}} }}^{2} } \\ \end{array} } \right]$$ where $\upsigma_{{{\rm{a}}_{\rm{i}} }}^{2}$ and $\upsigma_{{{\rm{a}}_{\rm{s}} }}^{2}$ are the variance of ${\mathbf{a}}_{{\mathbf{i}}}$, and ${\mathbf{a}}_{{\mathbf{s}}}$, respectively, $\upsigma_{{{\rm{a}}_{\rm{i}} {\text{a}}_{\rm{s}} }}$ is the covariance between sire effects for level and slope; the random pen and litter effects **p** and **l** were fitted as described in model (1); ${\mathbf{e}}$ is the vector of error terms, with Var(${\mathbf{e}}) = {\mathbf{I}} \otimes {\mathbf{R}}$, where ${\mathbf{R}}$ is the variance–covariance matrix, assumed to be independently normally distributed. Heterogeneous diagonal residual structures were also tested, but this was found to result in almost the same residual variance estimates and did not improve the model fit, as indicated by Akaike’s information criterion (AIC). ${\mathbf{X}}_{{{\mathbf{VL}}}}$ and ${\mathbf{Z}}_{{{\mathbf{VL}}}}$ are the incidence matrices for population average tolerance slope and those associated with each sire, respectively, consisting of the VL of each pig at the chosen stage of infection; ${\mathbf{X}}$ is the incidence matrix for the fixed effects, ${\mathbf{Z}}$ is the incidence matrix for the random sire effect on level; and ${\mathbf{U}}$ and ${\mathbf{M}}$ are the incidence matrices for pen and litter effects, respectively.

To test the significance of sire effects on level and slope, the model fit of the level-slope model was compared with that of nested models without any additive genetic effects (null model), and with only sire effects for level (level-model). Significance of each random effect was assessed using the likelihood ratio test (LRT) [35], with the LRT test statistics assumed to follow a $\chi^{2}$ distribution with a mixture of 0 and 1 degrees of freedom for the inclusion of sire effects only (e.g. null to level model, including sire effect) and a mixture of 1 and 2 degrees of freedom for the addition of sire slope effects and covariance (for example, from level to level-slope model) [36, 37].

### Estimation of the genetic variance in tolerance across stages of infection using a repeated measurement model

Next, to identify and estimate the genetic variance in tolerance across all stages of infection, ADG at the three stages of infection was considered as a repeated measure of the same trait and analyzed using the following univariate random regression sire model (level-slope repeated measures model):

3 $${\mathbf{y}} = {\mathbf{Xb}} + {\mathbf{X}}_{{{\mathbf{VL}}}} {\mathbf{b}}_{{\mathbf{s}}} + {\mathbf{Za}}_{{\mathbf{i}}} + {\mathbf{Z}}_{{{\mathbf{VL}}}} {\mathbf{a}}_{{\mathbf{s}}} + {\mathbf{Wc}}_{{\mathbf{i}}} + {\mathbf{W}}_{{{\mathbf{VL}}}} {\mathbf{c}}_{{\mathbf{s}}} + {\mathbf{Up}} + {\mathbf{Ml}} + {\mathbf{e}},$$

where ${\mathbf{y}}$ is the vector of repeated measures of ADG at early-, mid- and late-stages of infection (i.e. dimension 3 × number pigs considered in the study) and the incidence matrices ${\mathbf{X}}_{{{\mathbf{VL}}}}$, ${\mathbf{Z}}_{{{\mathbf{VL}}}}$ and ${\mathbf{W}}_{{{\mathbf{VL}}}}$ contain VL measures of individual pigs of the same stage of infection as the ADG-measurement in ${\mathbf{y}}$. The same fixed and genetic and additional random effects were fitted as described in model (2), with the addition of ${\mathbf{c}}_{{\mathbf{i}}}$ and ${\mathbf{c}}_{{\mathbf{s}}}$, denoting the vectors of permanent environmental effects for level and slope, respectively, for each individual, where $${\text{Var}}\left[ {\begin{array}{*{20}c} {{\mathbf{c}}_{{\mathbf{i}}} } \\ {{\mathbf{c}}_{{\mathbf{s}}} } \\ \end{array} } \right] = {\mathbf{I}} \otimes {\mathbf{C}}$$, with $${\mathbf{C}} = \left[ {\begin{array}{*{20}c} {\upsigma_{{{\rm{c}}_{\rm{i}} }}^{2} } & {\upsigma_{{{\rm{c}}_{\rm{i}} {\text{c}}_{s} }} } \\ {\upsigma_{{{\rm{c}}_{\rm{i}} {\text{c}}_{s} }}^{2} } & {\upsigma_{{{\rm{c}}_{s} }}^{2} } \\ \end{array} } \right]$$, where $\upsigma_{{{\rm{c}}_{\rm{i}} }}^{2}$ and $\upsigma_{{{\rm{c}}_{\rm{s}} }}^{2}$ are the variance of ${\mathbf{c}}_{{\mathbf{i}}}$, and ${\mathbf{c}}_{{\mathbf{s}}}$, respectively, and $\upsigma_{{{\rm{c}}_{\rm{i}} {\text{c}}_{\rm{s}} }}$ is the covariance between permanent environmental effects for level (overall mean at centred VL) and slope; with respective incidence matrices **W** and **W**_**VL**_. As described above, evidence for significant genetic variation in tolerance was obtained by comparing the model fit with that of the corresponding null and level repeated measure models using the LRT.

### Association of the WUR genotype with growth, resistance and tolerance

Associations of the WUR genotype with growth and resistance at each stage of infection were estimated by including the WUR genotype (coded as − 1, 0, 1 for *AA*, *AB* and *BB*, respectively) as fixed covariate in model (1). Similarly, associations of the WUR genotype with level and tolerance at each stage or across all stages of infection was assessed by including WUR genotype and WUR genotype-by-VL as additional fixed covariates in models (2) and (3). Thus, a statistically significant WUR genotype-by-VL interaction indicates genotypic differences in tolerance. Significance of the associations of the WUR genotype with traits was assessed based on Wald test statistics, where p < 0.05 was the significance threshold, as well as the LRT. The additive variance explained by the SNP was calculated as $2pq\alpha^{2}$, where $p$ and $q$ refer to the frequencies of *A* and *B* alleles in the population, respectively, and the allele substitution effect $\alpha$ for the trait in consideration was obtained from the corresponding models above. In this dataset, $p$ and $q$ were equal to 0.82 and 0.18, respectively, and the corresponding observed proportion of pigs with *AA*, *AB* and *BB* genotypes were 0.68, 0.28 and 0.04.

## Results

### Genetic parameters and relationship between ADG and VL across stages of infection

ADG for individual stages of infection ranged from − 110 to 800 g/day, with the minimum (− 110 g/day) and maximum ADG (800 g/day) observed at the early- and late-stages of infection, respectively (Table 2). Mean ADG increased with stage of infection from 250 to 430 g/day at early- and late-stages of infection, respectively, indicating that individuals were, generally, able to grow faster at later stages of infection. Mean VL increased from 37.1 to 67.6 units from the early- to the late-stage of infection. This apparent increase does not reflect a higher virus load at a particular time point during the corresponding stages, but is primarily due to the longer time-period associated with the late-stage of infection (on average 14.3 days compared to 6.9 and 9.2 days for the early- and mid-stages of infection, respectively). The coefficient of variation for growth rate was largest at the mid-stage of infection, whereas for VL it was approximately stable across stages (Table 2).

**Table 2:** Descriptive statistics of growth (ADG) and viral load (VL) at different stages of infection

Trait	Mean	Phenotypic Standard deviation	Minimum	Maximum
ADG_early_	250	10	− 110	640
ADG_mid_	290	10	− 90	680
ADG_late_	430	20	0	800
VL_early_	37.1	6.4	16.4	57.2
VL_mid_	58.4	9.5	35.6	87.1
VL_late_	67.6	10.7	39.1	99.3

ADG is measured in g/day and VL in AUC(log10 RT-PCR). Stages of infection are early-, mid-and late-stages

Both ADG and VL were found to be moderately heritable for each stage of infection (Table 3). Heritability of VL was highest at the mid-stage (between peak viremia and max viremia clearance (0.31)). Conversely, the heritability of ADG gradually decreased over subsequent stages of infection (from 0.28 to 0.16).

**Table 3:** Genetic components of growth (ADG) and viral load (VL) at different stages of infection

Trait	ADG_early_	ADG_mid_	ADG_late_	VL_early_	VL_mid_	VL_late_
ADG_early_	*0.28 (0.07)*	0.35 (0.03)	0.23 (0.03)	0.03 (0.03)	− 0.06 (0.03)	− 0.10 (0.03)
ADG_mid_	0.70 (0.14)	*0.22 (0.06)*	0.36 (0.03)	0.03 (0.03)	− 0.08 (0.03)	− 0.13 (0.03)
ADG_late_	0.77 (0.20)	0.84 (0.15)	*0.16 (0.08)*	− 0.05 (0.03)	− 0.20 (0.03)	− 0.19 (0.03)
VL_early_	0.06 (0.23)	0.73 (0.24)	− 0.02 (0.27)	*0.17 (0.10)*	0.49 (0.03)	− 0.28 (0.03)
VL_mid_	− 0.09 (0.18)	0.23 (0.20)	− 0.44 (0.20)	0.82 (0.08)	*0.31 (0.12)*	0.52 (0.02)
VL_late_	− 0.41 (0.23)	− 0.36 (0.22)	− 0.74 (0.21)	0.09 (0.26)	0.75 (0.15)	*0.21 (0.09)*

Estimates of heritability of growth (ADG) and viral load (VL) are shown at early-, mid- and late-stages of infection (on the diagonal), and of genetic and phenotypic correlations between the traits (lower and upper off-diagonals, respectively). Phenotypic variance was calculated by summing animal, litter and pen within-trial variance components. Heritability was calculated by dividing the animal variance component by the phenotypic variance. Standard errors are in parentheses. Variance component estimates for the non-genetic random effects (from the corresponding univariate models) are in Additional file 1: Table S2

Estimates of genetic correlations between stages of infection were generally high and positive for ADG (in the range from 0.70 to 0.84), while phenotypic correlations were also positive but weaker (Table 3). The bivariate models estimated genetic correlations for VL to be strong between consecutive stages of infection (0.75 to 0.82), but close to zero between the early- and late-stages (0.01), indicating that individuals genetically predisposed to experience lower VL early in infection did not necessarily have lower VL at the late stage. Phenotypic correlations for VL were moderate to strong between consecutive stages, but weak and negative between the early- and late-stages. Estimates of genetic correlations between ADG and VL varied in magnitude and sign depending on stage of infection, and had high standard errors. At the early- and mid-stages of infection, genetic correlations between ADG and VL were weakly to moderately positive (0.06 and 0.23 for early- and mid-stages, respectively). The strongest genetic correlation between VL and ADG (0.73 ± 0.24)) was observed for ADG at mid-stage of infection with VL at early-stage of infection, implying that individuals with higher genetic resistance for the early-stage of infection tended to experience a temporary reduction in growth after peak viremia. However, by the late-stage of infection, the genetic correlation between ADG and VL became strong and negative (− 0.74 ± 0.21), indicating that individuals whose genetic resistance led to lower VL at the clearance stage of infection tended to grow faster during that period. Phenotypic correlations between ADG and VL were typically weak across all stages of infection.

### Evidence for genetic variation in tolerance at each stage of infection

The null reaction-norm model [i.e. model (2)], which included VL as a fixed covariate but no genetic effects, identified a statistically significant linear association between growth and VL when late-stage ADG was regressed on mid-stage VL (p = 0.02) and when late-stage ADG was regressed on late-stage VL (p < 0.0001). No significant association was found between early-stage VL and growth at any stage of infection. As such, only models that included covariates for mid- and late-stage VL, respectively, are considered from hereon.

In the null models, population averages for tolerance (slope) were negative, but generally very flat (close to 0): for mid- and late-stage ADG on mid-stage VL, growth rate only decreased on average by − 1.4 (± 0.47) and − 2.4 (± 0.5) g/day per unit increase in VL, respectively; for late-stage ADG on late-stage VL, the decrease in growth rate was − 1.9 (± 0.4) g/day per unit VL increase. The log-likelihood of the model significantly increased when genetic effects (random sire effects) were included in the model (level-only model) for all stages of infection (p < 0.0001), indicating significant genetic variance in growth rate of pigs infected with PRRSV. Indeed, heritability estimates in the level-only reaction norm models (not shown) were similar to the corresponding univariate models. Models that included sire effects for both level and tolerance slope and a genetic covariance between them, also yielded a significantly better fit than the null model for all stages of infection (p < 0.0001). However, a statistically significant improvement of model fit of the level-slope model over the level model (i.e. evidence for significant genetic variation in tolerance) was only observed when late-stage ADG was regressed on mid-stage VL (p < 0.05) (Table 4, italic figures).

**Table 4:** Estimates of genetic variance components for ADG (g/d) regressed on VL

VL	ADG	Level-only model		Level-slope model				Model fit
		Level	Residual	Level	Covariance	Slope	Residual	P value
Mid	Mid	3.47 (1.56)	11.10 (0.61)	3.39 (1.56)	~0 (~ 0)	3.40E−03 (6.24E−03)	11.00 (0.62)	0.74
	Late	2.33 (1.24)	11.9 (0.67)	2.52 (1.30)	− 6.90E−02 (9.61E−02)	*1.64E*−*02 (1.02E*−*02)*	11.60 (0.67)	*0.02*
Late	Late	1.96 (1.15)	12.00 (0.68)	1.97 (1.15)	~0 (~ 0)	~0 (~ 0)	12.00 (0.68)	1.00

ADG at mid- and late-stage of infection is regressed on VL at mid- and late-stage of infection. The last column denotes the p-value of the LRT used to test whether the level-slope model significantly improves the model fit over the level model. For definition of stages of infection, see text. Standard errors are in brackets. Estimates under 1E−07 have been reported as close to 0 (~ 0)

Estimates of genetic variance for level were relatively stable across stages of infection for both the level-only model and the level-slope model (Table 4). However, estimates of genetic variance for tolerance slope differed greatly between stages of infection, by up to four orders of magnitude (Table 4). As expected, genetic variance for slope was largest when late-stage ADG was regressed on mid-stage VL 16.4(± 10.2). The corresponding genetic covariance between level and tolerance slope was not significantly different from zero.

### Genetic variation for tolerance across all stages of infection: repeated measure model

The average tolerance slope estimated by the repeated measures null-model was − 0.46 (± 0.28) g/day per unit VL decrease and not significantly different from zero (p = 0.35). BW at the start of infection ranged from 3.72 to 13.43 kg, with an average of 7.38 kg. A weak but significant association of BW at the start of infection with growth under infection was found (p < 0.0001), with a positive regression coefficient of 19.0 (± 2.83) g/day per kg difference in BW. In other words, pigs that were 1 kg heavier at the beginning of the challenge experiment tended to grow on average 19 g/d faster during the 42-day infection period. The log-likelihood of the repeated measures model improved significantly when genetic effects for level (i.e. random sire effects) were included in the model (level model) (p < 0.0001) (Table 5). Furthermore, a statistically significant increase in the log-likelihood over the level model was observed when genetic effects for level, slope and a covariance between the latter were included (p < 0.0001), indicating significant genetic variation in tolerance across all stages of infection.

**Table 5:** Genetic and permanent environmental variance components for ADG (g/d) obtained by the repeated measures model

	Null repeated measure model	Level-only repeated measure model	Level-slope repeated measure model
Genetic			
Level		2.16 (0.91)	2.89 (1.16)
Covariance			0.103 (0.054)
Slope			0.012 (0.003)
PEV			
Level	2.01 (0.34)	2.01 (0.34)	2.89 (1.16)
Covariance	0.03 (0.01)	0.03 (0.01)	0.103 (0.005)
Slope	0.002 (0.001)	0.003 (0.001)	0.01 (0.003)
Other			
Pen	0.62 (0.20)	0.66 (0.20)	0.66 (0.20)
Litter	1.21 (0.29)	0.70 (0.26)	0.70 (0.27)
Residual	10.51 (0.39)	10.49 (0.39)	10.15 (0.38)
Log Likelihood	4846.7	4855.49	4884.44

Estimates include ADG and VL measures for all three defined stages of infection (early, mid and late), together with the Log-Likelihood value associated with the different models. Variance components estimated from random regression models: null model, containing no genetic effect; level-only model, containing only the overall sire effect on growth under infection; and level-slope model, containing sire effects on level and slope, respectively. All other fixed effects/covariates and random effects were identical between models. Variance component estimates for the other random effects (e.g. pen, litter, common environmental, residuals) were identical between the models and not shown. Standard errors are in brackets

### Association of the WUR genotype with tolerance at individual and across stages of infection

The WUR genotype was associated with VL at each individual stage of infection (p < 0.0001), but was only significantly associated with ADG at mid- and late-stages of infection (p < 0.0001). Individuals carrying the beneficial *B* allele at the WUR SNP generally had lower VL and higher ADG than *AA* animals. The largest difference in ADG between genotypes was at the late-stage of infection, where *AB* individuals grew, on average 45 g/day faster than *AA* animals. However, the largest difference in VL occurred at the mid-stage of infection, where *AB* animals had on average 3.6 AUC units lower VL than *AA* animals.

The WUR genotype explained 43.3 and 12.3% of the genetic variance of early- and mid-stage VL, respectively, but only 4.4% of the genetic variance of late-stage VL. In contrast, it had no significant effect on early-stage ADG, but explained 19.4 and 19.7% of the genetic variance of mid- and late-stage ADG, respectively. Importantly, the WUR genotype had no significant effect on tolerance at any stage of infection as indicated by non-significant genotype-by-VL interactions (p = 0.80, 0.20 and 0.37 for early-, mid- and late-stages of infection, respectively).

Based on the repeated measures model, the WUR genotype was significantly associated with VL and ADG across all stages of infection. *AB* individuals grew, on average, 40 g/day faster than *AA* animals and had on average 3.8 AUC units lower VL. In contrast to analysis of the individual stages of infection, the WUR genotype was also significantly associated with tolerance based on the repeated measures model across all stages of infection (p = 0.004), where genotypic differences in tolerance were indicated by the significant genotype-by-VL interaction [9]. As with resistance, the *B* allele also conferred higher tolerance. Individuals with the *AB* genotype grew on average by 10 g/d more per unit increase in VL than individuals with the *AA* genotype.

## Discussion

### Summary of findings

Although numerous studies have left little doubt that pigs vary genetically in both resistance to, and growth under, PRRSV infection [6, 10, 24, 26, 31, 34], evidence for genetic variation in tolerance of pigs to PRRS has thus far been inconclusive [10, 13]. This study provides, for the first time, evidence for significant genetic variation in tolerance of pigs to PRRS. This was obtained by partitioning an individual’s infection into three distinct phases based on individual viremia profile characteristics, instead of considering resistance and performance as single cumulative measures over a 21- or 42-day infection period, as was the case in previous analyses [6, 10, 24, 31]. This partitioning helped to resolve the previously encountered statistical constraints for detecting genetic variation in tolerance of pigs to PRRS in two ways: first, it provided repeated measurements of virus load and growth to boost the statistical power of the reaction-norm models covering the entire 42-day infection period, and second, it helped to focus the analysis to a stage of infection where genetic differences in tolerance are most pronounced.

Growth and resistance were heritable across all stages of infection, which implies that genetic selection for resistance to PRRS is expected to affect growth and viremia at all stages of infection. Estimates of heritability and genetic variance for resistance were greatest for VL during the highly immune-active time-period between peak viremia and maximal rate of viremia clearance [38]. This suggests that the phase associated with the most rapid viremia decline would be the most affected by genetic selection. For growth, heritability was highest at the early stage of infection. It is not clear whether this reflects heritable genetic differences in growth per se, or already captures different growth responses to infection. Genetic correlation between resistance and growth was strongest between pre-peak VL and post-peak ADG, which may indicate that growth response lags behind and may be affected by earlier viremia response. Other studies on pigs also observed that estimates of genetic associations between growth and some immune response traits become stronger when growth was recorded at a later stage than immune response measures, although this also depended on the type of immune measure considered [39].

With the univariate single-stage models, significant genetic variance in tolerance was only identified when late-stage ADG was regressed on mid-stage VL. This indicates that genetic variation in tolerance may be sensitive to the timing of measurements. In this case, a unit reduction in VL at the rapid phase of post-peak viremia decline corresponded to genetic differences in pig growth responses at the late-stage of infection, when virus load is low. Indeed, where some pigs may be genetically predisposed to experience compensatory growth, other pigs may be predisposed to suffer prolonged growth depression [40].

Adopting a repeated measurements model that included viremia and growth measures at the different stages of infection, provided further evidence for genetic variation in tolerance, as indicated by the considerably better model fit of the level-slope model over the level-only model. According to this model, genetic improvement of one genetic standard deviation in tolerance would correspond to a 3.5 g/day difference in growth per unit change in VL (Table 5). This means that pigs with equal growth rate at mean VL (57.5 units of VL, at which the repeated measures model for tolerance was centred), but one genetic standard deviation higher tolerance are expected to grow 159 g/d faster at the observed maximum VL of 99.3 VL units. In other words, pigs that have low resistance and, therefore, have high VL, would benefit from a genetically higher level of tolerance. It would be interesting to know whether such gain in tolerance is compromised by lower growth in the absence of infection. Comparable performance measures of the same pigs prior to infection or of non-infected relatives would help resolving this question [10].

### Associations of the WUR genotype with growth, resistance and tolerance

In line with previous analyses of the same data, we found a significant and similar effect of genotype at the WUR SNP for both VL and ADG, where individuals with the favourable *AB* genotype experienced lower VL and higher ADG [24, 26]. As in previous studies, we do not report the results with the *BB* genotype due to the small number of individuals (n = 36). Partitioning the infection period into distinct phases, further revealed that the WUR genotype had the strongest effect on VL at early- to mid-stages of infection. These results complement the findings of Boddicker et al. [24], who identified a 13.3% reduction in VL between 0 and 21 dpi for individuals with the favourable *AB* genotype. Furthermore, these results agree with those of Hess et al. [26] based on the same data, who found that the *AB* genotype was associated with lower and earlier peak viremia and faster post-peak viremia decline. In contrast, the WUR genotype effect on growth was not significant at the early-stage of infection, but strong (explaining almost 20% of the genetic variance in growth) at the stages of post-peak viremia decline, highlighting a possible lag effect of WUR on growth as a by-product of its stronger association with VL at earlier stages. Boddicker et al. [24] previously identified a 9.1% increase in growth in individuals with the *AB* genotype. This was identified for the entire period of infection (0–42 dpi), which may have reduced the genetic signal due to the lag effect of VL on ADG. This may explain why a higher increase in growth for individuals with the *AB* genotype was found in this study (19.4 and 19.7% for mid- and late-stages of ADG, respectively).

The effect of WUR genotype on tolerance slope was not statistically significant at any single stage of infection, which either indicates lack of statistical power or suggests that the association between WUR and ADG at mid- to late-stage of infection is a direct consequence of the effect of the WUR genotype on resistance. However, the repeated measurements model identified a statistically significant association between WUR genotype and overall tolerance slope across all stages of infection, with the beneficial *B* allele not only conferring greater resistance but also greater tolerance. This indicates that resistance and tolerance, when monitored over a prolonged infection period, may share common genetic pathways that are partly influenced by the WUR SNP. One common pathway may be guanylate binding protein 5 (GBP5), the putative causative mutation for the observed associations of WUR, which was found to be in complete linkage disequilibrium with the WUR SNP [41]. GBP5 is associated with innate immune response to infection, where *AA* animals lack GBP5 functionality [41]. As such, it has been hypothesised that reduced viral replication (and thus reduced viral load) in *AB* animals is due to the functionality of GBP5. However, the function of GBP5 with respect to tolerance mechanisms remains unknown. Given the association of the WUR SNP with both resistance and tolerance, as identified in this study, it is possible that GBP5 may also play a role in tolerance.

### Benefits arising from partitioning the infection period into different stages of infection

One of the novelties of this study compared to previous analyses of the same dataset was the partitioning of the infection period into distinct viremia-profile phases. This resulted in two noticeable benefits: first, it provided repeated measurements for virus load and growth that boosted the statistical power of the random regression models, which was key for detecting genetic variation in tolerance of pigs to PRRS in this study. Second, partitioning the infection period allowed for the possibility that genetic mechanisms for resistance and tolerance differ over the time course of infection and provided the ability to focus on particular stages of infection where genetic differences in resistance or tolerance may be most pronounced. Using cumulative viremia over a prolonged period of time (e.g. over 21 or 42 days) provides a useful summary measure of an individual’s ability to cope with infection, but it does not capture the dynamic changes of the individual’s viremia curve that could result from the action of different immune functions at different stages of infection. For example, two individuals may have equal VL over the 21-day infection period, but one of the individuals may experience high viremia over a short time followed by rapid clearance, while the other may have moderate viremia over a prolonged time [34]. This may reflect different immune response patterns and may yield a blurred genetic signal when cumulative VL is used as phenotype [42]. By defining stages of infection based on underlying viremia curve characteristics, the corresponding VL measures may reflect the effect of different immune response mechanisms to PRRSV acting over time, and thus possibly different sets of genes associated with these. For example, the time of fastest rate of viremia clearance, which defined the boundary between the mid- and late-stage of infection in this study, may coincide with the time at which neutralizing antibodies start to be produced [43]. Thus, the mid- to late-stages of infection, for which significant genetic variance in both resistance and tolerance were identified in this study, are likely determined by the ability of the individual to mount an effective adaptive immune response [44] and how it deals with the associated implications on growth [45]. Indeed, previous studies found considerable genetic variation in total antibody response (measured by serum IgG levels) of pigs to PRRSV at 6 weeks post-infection [46, 47].

Partitioning the infection period into distinct phases also revealed that genetic parameters for resistance, tolerance, and growth changed considerably over the time-course of infection. For example, the estimate of the genetic correlation was strongly positive between mid-stage ADG and early-stage VL (0.84 + 0.15), indicating that genetically resistant individuals with lower VL earlier in infection have slower growth rate in the subsequent stage of infection. These results are consistent with resource allocation theory, where a temporary trade-off between fighting infection and growth may occur due to limited nutrient resources being available [45, 48, 49]. At the late-stage of infection, the genetic correlation between ADG and VL shifted from strongly positive to negative (i.e. − 0.74 + 0.21 between late-stage VL and late-stage ADG). This period is associated with return to homeostasis or even compensatory growth, where animals that managed to clear the virus faster can allocate resources to growth [50]. In summary, these results imply that selection for lower VL at the pre-peak viremia phase may lead to a reduction in growth during that phase but in faster growth at later stages of the infection period, and overall. Furthermore, our results suggest that genetic selection to increase tolerance would be effective only in the phase of rapid viremia decline.

### Sensitivity of tolerance estimates to the partitioning of the infection period

In this study, stages of infection were defined with the help of the mathematical Woods function, which allowed partitioning into distinct infection phases based on viremia profile characteristics, i.e. the phase associated with viremia increase towards peak viremia, the phase of rapid post-peak decline, and the later phase corresponding to more gradual viremia decline. The question arises on how sensitive the results are to the definition of infection stages according to the Woods function. Previous analyses of the viremia data from trials 1 to 8 had fitted a LOESS curve through the data and integrated to obtain area under the curve from 0 to 21 dpi [26, 34]. A subsequent study demonstrated similar heritabilities and high genetic and phenotypic correlations between viral loads established by both methods, and concluded that both methods describe the same biological trait [34]. Furthermore, the analyses of this study were repeated for an alternative definition of stages of infection, in which they were simply defined according to the following three fixed time-periods: from 0 to 7 dpi (early), from 7 to 14 dpi (mid) and from 14 to 42 dpi (late). As shown in Additional file 2, the fixed time-periods provided similar evidence for genetic variation in tolerance: the repeated measurement model identified significant genetic variation in tolerance to infection and the single measurement models applied to VL and ADG at different stages of infection identified genetic variance in tolerance only for the late-stage of infection (although in this case when late-stage ADG was regressed on late-stage VL, rather than on mid-stage VL, as was the case when stages of infection were defined based on viremia characteristics). These results indicate that specification of infection stages according to individual profile characteristics is not essential for detecting genetic variation in tolerance. Nevertheless, time-periods that are based on individual infection profiles rather than on fixed measurement times would be expected to better reflect the dynamic behaviour of genetic resistance and tolerance mechanisms, as indicated by generally higher heritability estimates for VL and ADG (see Additional file 2). Finally, it should be mentioned that partitioning data into distinct stages of infection has proven useful, but may not harness the full information about how resistance and tolerance mechanisms interact over time that is captured by the longitudinal viremia and growth data. Such information may be better portrayed through individual infection trajectories [11, 12, 51], although the methodology for genetic trajectory analyses is still in its infancy.

### Implications for breeding programmes and conclusions

The results of this study suggest that, in principle, genetic selection for greater tolerance of pigs to PRRS is possible and may indeed result in an increase in growth rate of pigs infected with PRRSV. However, in practical terms tolerance may be difficult to target for genetic selection, given that multiple repeated measures may be needed for each individual, and information of performance and within-host–pathogen load are needed for multiple offspring of each sire. This high demand in data for estimating tolerance suggests that genetic improvement of tolerance of pigs to PRRSV can only be achieved realistically with genomic selection and intense recording schemes that involve repeated measurements to reliably estimate genetic effects [52]. Furthermore, incorporation of tolerance into breeding programmes would require understanding the genetic correlations between resistance and tolerance, and how these both contribute to overall resilience of pigs exposed to PRRSV [15]. Note that we were not able to estimate genetic correlations between resistance and tolerance to PRRS in this study because VL was used both as a measure of resistance (i.e. response variable) and as predictor for tolerance in the random regression models. This sequential interdependence calls for more sophisticated methods such as structural equation models to estimate the true relationships between these traits [53, 54].

Perhaps the most relevant result of this study regarding practical pig breeding was that the genotype at the WUR SNP was found to be significantly associated with resistance, growth and tolerance, with the favourable *B* allele likely improving all three traits. This suggests that resistance and tolerance are partly pleiotropic and thus work together to improve host performance, and that selection for greater resistance may simultaneously improve tolerance, and consequently also growth of piglets infected with PRRSV. Based on the results of this study, consideration of the WUR genotype in genetic selection schemes appears to be a promising strategy to improve simultaneously resistance and tolerance of growing pigs to PRRS in the absence of intense data recording.

## Additional files

**Additional file 1: Table S1.** Animal composition of the data from the PHGC trials used in this study. **Table S2.** Variance components for ADG (g/d) and VL at early, mid and late stages of infection.

**Additional file 2.** Assessing the sensitivity of model results to different definitions of ‘stages of infection’. Results are shown when stages of infections are defined by fixed time-periods rather than according to viremia characteristics.
