# Supplementary material for: Harnessing longitudinal information to identify genetic variation in tolerance of pigs to Porcine Reproductive and Respiratory Syndrome virus infection
Source: Genet Sel Evol. 2018 Oct 24;50:50. doi: 10.1186/s12711-018-0420-z (PMC6201485; doi:10.1186/s12711-018-0420-z)
Supplement: Supplementary file 1 — Additional file 1: Table S1. Animal composition of the data from the PHGC trials used in this study. Table S2. Variance components for ADG (g/d) and VL at early, mid and late stages of infection. [file 12711_2018_420_MOESM1_ESM.docx]

**Table S1 Animal composition of the data from the PHGC trials used in this study**

| Trial | Number of animals | Number of sires | Number of dams | Average litter size | Breed cross | Genetic background^a^ |
| --- | --- | --- | --- | --- | --- | --- |
| 1 | 117 | 4 | 62 | 1.89 | LW × LR | A |
| 2 | 123 | 9 | 64 | 1.92 | LW × LR | A |
| 3 | 83 | 6 | 38 | 2.18 | LW × LR | A |
| 4 | 137 | 5 | 33 | 4.15 | Duroc × LW/LR | B |
| 5 | 134 | 8 | 32 | 4.19 | Duroc × LR/LW | C |
| 6 | 32 | 2 | 15 | 2.13 | LR × LR | D |
| 7 | 170 | 6 | 27 | 6.30 | Pietrain × LW/LR | E |
| 8 | 107 | 7 | 32 | 3.35 | Duroc × LW/LR | F |
| 15 | 108 | 7 | 35 | 3.09 | Pietrain × LW | G |

LW: large white breed; LR*:* landrace breed

^a^ Genetic background is defined as pigs from the same breeding company and the same breed cross

**Table S2 Variance components for ADG (g/d) and VL at early, mid and late stages of infection**

| **Variance** | **ADG_early_** | **ADG_mid_** | **ADG_late_** | **VL_early_** | **VL_mid_** | **VL_late_** |
| --- | --- | --- | --- | --- | --- | --- |
| **Genetic** | 3.69 (0.96) | 3.06 (0.92) | 2.63 (1.33) | 6.72 (4.12) | 28.39 (11.84) | 23.68 (11.22) |
| **Pen** | 1.13 (0.32) | 0.79 (0.28) | 2.26 (0.50) | 0.93 (0.61) | 0 (0) | 8.74 (2.56) |
| **Litter** | 0 (0) | 0 (0) | 0.27 (0.66) | 4.70 (2.02) | 4.23 (4.84) | 5.30 (5.10) |
| **Residual** | 8.49 (0.81) | 9.87 (0.83) | 11.40 (0.94) | 28.10 (2.68) | 57.79 (7.01) | 77.50 (7.36) |
| **Phenotypic** | 13.30 (0.62) | 13.72 (0.61) | 16.52 (0.78) | 40.44(1.84) | 90.40 (4.26) | 115.21 (5.27) |
| **h^2^** | **0.28 (0.07)** | **0.22 (0.06)** | **0.16 (0.08)** | **0.17 (0.10)** | **0.31 (0.12)** | **0.21 (0.09)** |

*Estimates were obtained from the univariate models [1], ADG is measured in g/day and VL in AUClog10RT-PCR. Stages of infection are early, mid and late stages. Standard errors are in brackets.*
