# Supplementary material for: Harnessing longitudinal information to identify genetic variation in tolerance of pigs to Porcine Reproductive and Respiratory Syndrome virus infection
Source: Genet Sel Evol. 2018 Oct 24;50:50. doi: 10.1186/s12711-018-0420-z (PMC6201485; doi:10.1186/s12711-018-0420-z)
Supplement: Supplementary file 2 — Additional file 2. Assessing the sensitivity of model results to different definitions of ‘stages of infection’. Results are shown when stages of infections are defined by fixed time-periods rather than according to viremia characteristics. [file 12711_2018_420_MOESM2_ESM.docx]

**Assessing the sensitivity of model results to different definitions of ‘stages of infection’**

**Period of infection defined by duration**

In the main text, we defined stages of infection by individual viremia profile characteristics to identify genetic variation in tolerance at each stage of infection, and across all stages of infection using a repeated measures model. In this appendix, we used fixed time intervals as a proxy for different periods of infection. Periods of infection were defined from 0 to 7 dpi (early), 7 to 14 dpi (mid) and 14 to 42 dpi (35 dpi for trials 7 and 8) (late), as these durations were similar to the average viremia peak, and maximal rate of viremia clearance used to define stages of infection in the main text. It should be noted, that although previous research had used up to 21 dpi to define acute stage of infection, 14 dpi was closer to maximal rate of viremia clearance, so was used to define infection periods here. These duration-defined periods of infection were implemented using model [2] to identify genetic variation at each period of infection and in repeated measures model [3] to identify genetic variation in tolerance across all periods (see main text).

**Genetic parameters of ADG and VL at period of infection**

Table A1 below shows estimates of heritability of ADG and VL at each infection period. For ADG, heritability decreased over the time course of infection, where the estimate was highest in the 0 to 7 dpi infection period (0.27+0.12), and lowest at the 14 to 42 dpi infection period (0.15+0.06). Conversely, estimates of heritability of VL remained stable over the time course of infection.

**Table A1**. **Estimates of heritability of growth (ADG) and viral load (VL) at each period of infection.**

| **Trait** | **Duration** | **h^2^** |
| --- | --- | --- |
| **ADG** | 0 - 7 | 0.27 (0.12) |
|  | 7 – 14 | 0.17 (0.09) |
|  | 14 – 42 | 0.15 (0.06) |
| **VL** | 0 - 7 | 0.20 (0.10) |
|  | 7 – 14 | 0.17 (0.09) |
|  | 14 – 42 | 0.22 (0.11) |

*Each period of infection is defined by fixed time duration from 0 to 7, 7 to 14 and 14 to 42 dpi. Standard errors are in brackets.*

**Genetic variation in tolerance at different periods of infection**

The null model including VL as a fixed covariate identified a statistically significant linear association between growth and VL (p<.0001). The log-likelihood of the model was significantly improved when genetic effects (random sire effects) were included in the model (level model) at all periods of infection (p<.0001), indicating significant genetic variance in growth of pigs infected with PRRSV. However, as indicated in Table A2, significant improvement of the level-slope model fit over the level-only model only occurred later in infection i.e. in the 14-42 dpi infection period.

**Table A2**. **Genetic variance components for ADG (g/d) at each period of infection.**

|  |  |  |  |  |  |  |
| --- | --- | --- | --- | --- | --- | --- |
|  | | **Level-only model** | **Level-Slope model** | | | **Model fit** |
| **VL** | **ADG** | **Level** | **Level** | **Covariance** | **Slope** | **P-value** |
| **0-7** | **0-7** | 2.82  (1.41) | 2.84  (1.42) | 0.05  (0.16) | 5.39E-03  (0.03) | 0.87 |
|  | **7-14** | 3.76  (2.06) | 3.71  (2.07) | -0.25  0.28) | 0.03  (0.07) | 0.55 |
|  | **14-42** | 2.76  (1.36) | 2.76  (1.36) | ~0 | ~0 | 1.00 |
| **7-14** | **7-14** | 4.24  (2.18) | 4.24  (2.18) | ~0 | ~0 | 1.00 |
|  | **14-42** | 2.93  (1.36) | 2.86  (1.37) | -0.04  0.17 | 0.03  (0.05) | 0.77 |
| **14-42** | **14-42** | 2.04  (1.10) | 2.90  (1.40E-03) | 1.02E-04  (6.02E-05) | **8.53E-03**  **(3.60E-03)** | **0.001** |

*Periods of infection are defined by fixed time periods from 0 to 7, 7 to 14 and 14 to 42 dpi. The last column denotes the p-value of the LRT used to test whether the level-slope model significantly improves the model fit over the level model. For definition of stages of infection, see main text. Standard errors are in brackets.*

**Genetic variation in tolerance across all periods of infection**

When the repeated measurement model was applied, the level-slope model was a significantly better fit than the level-only model (p<.0001) (Table A3), indicating genetic variation in tolerance across all periods of infection could also be detected when periods of infection were defined by fixed time periods rather than by time periods defined by viremia curve characteristics.

**Table A3**. **Variance components for ADG (g/d) over all duration-defined periods of infection.**

|  | **Null repeatability model** | **Level repeatability model** | **Level-Slope repeatability model** |
| --- | --- | --- | --- |
| **Level** | . | 2.36  (0.91) | 2.84  (1.08) |
| **covariance** | . | . | 0.03  0.02 |
| **Slope** | . | . | 1.23E-03  (4.20E-04) |
| **Residual** | 16.20  (0.45) | 16.20  (0.44) | 11.57  (0.43) |
| **LogLikelihood** | **4499.17** | **4510.58** | **4530.61** |

*Infection periods are defined from 0 to 7, 7 to 14 and 14 to 42 dpi. Standard errors are in brackets.*

**Association of WUR genotype with tolerance slope**

The WUR was not associated with tolerance slope at any stage of infection or across all stages of infection (p>0.05).
